# High A20 expression negatively impacts survival in patients with breast cancer

**DOI:** 10.1371/journal.pone.0221721

**Published:** 2019-08-26

**Authors:** Chang Ik Yoon, Sung Gwe Ahn, Soong June Bae, Yun Jin Shin, Chihwan Cha, So Eun Park, Ji-Hyung Lee, Akira Ooshima, Hye Sun Lee, Kyung-Min Yang, Seong-Jin Kim, Seok Hee Park, Joon Jeong

**Affiliations:** 1 Department of Surgery, St Mary’s Hospital, Catholic University College of Medicine, Seoul, Korea; 2 Department of Surgery, Gangnam Severance Hospital, Yonsei University College of Medicine, Seoul, Republic of Korea; 3 Gangnam Severance Hospital, Yonsei University College of Medicine, Seoul, Republic of Korea; 4 Department of Biological Sciences, Sungkyunkwan University, Suwon, Gyeonggi-do, Republic of Korea; 5 Precision Medicine Research Center, Advanced Institutes of Convergence Technology; 6 Biostatistics Collaboration Unit, Yonsei University College of Medicine, Seoul, Republic of Korea; 7 Department of Transdisciplinary Studies, Graduate School of Convergence Science and Technology, Seoul National University, Suwon, Kyunggi-do, Korea; The University of Newcastle, AUSTRALIA

## Abstract

**Background:**

A20 protein has ubiquitin-editing activities and acts as a key regulator of inflammation and immunity. Previously, our group showed that A20 promotes tumor metastasis through multi-monoubiquitylation of SNAIL1 in basal-like breast cancer. Here, we investigated survival outcomes in patients with breast cancer according to A20 expression.

**Patients and methods:**

We retrospectively collected tumor samples from patients with breast cancer. Immunohistochemistry (IHC) with an A20-specific antibody was performed, and survival outcomes were analyzed.

**Results:**

A20 expression was evaluated in 442 patients. High A20 expression was associated with advanced anatomical stage and young age. High A20 expression showed significantly inferior recurrence-free-survival and overall-survival (*P<*0.001 and *P*<0.001, respectively). Multivariate analysis showed that A20 was an independent prognostic marker for RFS (HRs: 2.324, 95% CIs: 1.446–3.736) and OS (HRs: 2.629, 95% CIs: 1.585–4.361). In human epidermal growth factor receptor 2 (HER2)-positive and triple negative breast cancer (TNBC) subtypes, high A20 levels were associated with poor OS.

**Conclusion:**

We found that A20 expression is a poor prognostic marker in breast cancer. The prognostic impact of A20 was pronounced in aggressive tumors, such as HER2-positive and TNBC subtypes. Our findings suggested that A20 may be a valuable target in patients with aggressive breast cancer.

## Background

Breast cancer is the most common cancer in women worldwide, and 1.5 million women are diagnosed each year. With the development of improved treatment modalities and new drugs, the survival rate has improved, and the annual breast cancer mortality rate has decreased by almost 2.3% [1].

Despite advances in systemic therapy, including targeted therapies, such as anti-estrogen and anti-human epidermal growth factor receptor-2 (HER2) therapies, treatment outcomes for metastatic cancer remain poor. To improve treatment outcomes for patients with breast cancer, there is an urgent need to identify novel targets that play an integral role in the metastatic cascade.

The A20 protein, which is also called tumor necrosis factor α-induced protein 3 (TNFAIP3), acts as a key regulator of inflammation and immunity since it stimulates anti-apoptotic signaling pathway through suppression of TNF signal pathway and regulates nuclear factor-κB signaling [2–5]. Recent studies have also shown that A20 protein has ubiquitin (Ub)-editing activities, including deubiquitylase [6–8] and E3 ubiquitin ligase activities [9], and can function as a ubiquitin-binding protein [10]. Although the roles of A20 in inflammation and immune responses are well understood, its functions in cancer are still unclear. However, several studies in tumor cell lines, such as hepatocellular carcinoma, glioma stem cells and breast cancer, have suggested that A20 plays a role in carcinogenesis by preventing apoptosis [11–14].

Recently, our group reported that the A20 protein plays an oncogenic role in mediating epithelial mesenchymal transition (EMT), which is strongly associated with cancer progression characteristics, such as invasion and metastasis [15]. Our study showed that A20 protein promoted metastasis in triple negative breast cancer (TNBC) through multi-monoubiquitylation of Snail1 [16]. We found, through *in vitro* and *in vivo* experiments, that the A20 protein was more highly expressed in aggressive tumors and that A20 upregulation promoted metastasis. However, the clinical characteristics of A20 expression in breast cancer have been poorly explored.

The aim of this study was to evaluate the association between A20 expression and survival outcomes in patients with breast cancer. We also investigated the prognostic impact of A20 expression according to immunohistochemistry (IHC)-based molecular subtypes.

## Patients and methods

### Patients

We retrospectively collected tumor tissues from patients undergoing surgery for breast cancer at Gangnam Severance Hospital in Seoul, Korea between January 1996 and December 2014. All the study subjects were diagnosed with stage I–III primary breast cancer. These patients underwent adjuvant treatments according to standard protocols. Clinical data were collected, including age at surgery, histologic grade (HG), lymph node status, estrogen receptor (ER) status, progesterone receptor (PR) status, HER2 status, lymphovascular invasion (LVI), Ki67 levels, treatment modality, breast cancer recurrence, and death.

TNM stage was determined according to the American Joint Committee on Cancer (AJCC), 7^th^ edition. Tumor grade was determined using the modified Scarff-Bloom-Richardson grading system. Before February 1999, ER and PR status were assessed using a ligand binding assay, and tumors were considered positive if the score was >10 fmol/mg.

The study protocol was approved by the institutional review board of Gangnam Severance Hospital.

### Ethics approval and consent to participate

Our study itself was conducted as human-specimen subject research and was approved by the institutional review board (IRB) review (Local IRB number: 3-2018-0067). All procedures performed in studies involving human participants were in accordance with the ethical standards of the institutional and/or national research committee and with the 1964 Helsinki declaration and its later amendments or comparable ethical standards. The need for informed consent was waived under the approval of the IRB due to the retrospective design.

### Immunohistochemistry (IHC) and molecular subtyping

As previously described [17], we evaluated ER, PR, HER2, and Ki67 expression using the following antibodies, ER (1:100 clone 6F11; Novocastra, Newcastle upon Tyne, UK), PR (clone 16; Novocastra), HER2 (4B5 rabbit monoclonal antibody; Ventana Medical Systems, Tucson, AZ, USA), and Ki-67 (MIB-1; Dako, Glostrup, Denmark). ER- and PR-positive IHC expression was defined according to the modified Allred system: positive, Allred score 3–8 and negative, Allred score 0–2. HER2 status was re-evaluated according to American Society of Clinical Oncology/College of American Pathologists guideline [18]. HER2 status was considered positive if the score was 3+, and was considered negative with a score of 0 or 1+. Tumors with a score of 2+ underwent FISH or SISH analysis, according to the manufacturer’s instructions (PathVysion kit; Vysis, Downers Grove, IL, USA or HER2 inform; Ventana). Ki-67 expression is presented as the percentage (range 0–100%) of positive tumor cells.

For the molecular subtyping, the following definitions were used: i) Luminal/HER2 negative: ER positive and/or PR positive and HER2 negative; ii) HER2 positive: HER2 positive regardless of ER and PR status; and iii) TNBC: ER negative, PR negative, and HER2 negative.

### Tissue microarray and IHC staining to evaluate A20 expression

Tissue microarray (TMA) paraffin blocks were generated as previously described using an Accu Max Array tissue-arraying instrument (Petagen, Inc., Seoul, Korea) [17]. For IHC, each TMA slide was stained with a rabbit monoclonal anti-A20 antibody (ab92324, 1:200; Abcam) and counter-stained with hematoxylin. After staining, A20 expression, assessed as cytoplasmic staining, was scored by an experienced pathologist (A.O.) using a microscope (400× magnification). Positive A20 expression in tumor cytoplasm was defined when the percentage of stained cells was equal to more than 60%. Finally, a score of 2+ or 3+ was defined as high A20 expression, whereas a score of 0 or 1+ was defined as low A20 expression (S1 Fig). The IHC results were interpreted blindly, without any information regarding clinical parameters or outcomes.

### Statistical analysis

Continuous variables were compared using Student’s t-test or Mann-Whitney test. Categorical variables were compared using the Chi-square test or Fisher’s exact test. Overall survival (OS) was defined as the period from primary curative surgery to the last follow-up or death from any cause. Recurrence free survival (RFS) was defined the period from primary curative surgery to the date of any recurrence (loco-regional or distant metastasis), death due to any cause or the last follow-up. Data for patients who did not have an event of interest were censored at the date of the last follow-up. Kaplan-Meier survival curves were compared using the log-rank test. Clinicopathologic factors associated with survival outcomes were analyzed using the Cox proportional hazard model. The variables used in the multivariate analysis were those that showed statistical significance in the univariate analysis for OS and RFS.

To compare the predictive power of the Cox proportional hazard model with and without A20, we applied the Harrell’s c-statistic to obtain concordance index (c-index) [19]. It is calculated to measure the concordance for time-to event data, in which increasing values between 0.5 and 1.0 indicated improved prediction. For model comparison, the bootstrapping method was used with resampling 1,000 times. We also performed net reclassification improvement (NRI) and integrated discrimination improvement (IDI) to evaluate improvement of discriminating ability when new factors were added in the survival model [20]. In NRI and IDI, a significant improvement is recognized in the prediction model when their values are greater than 0.

These analyses were performed using SPSS version 23 (SPSS; Chicago, IL, USA) and R version 3.4.2 (www.R-project.org). Statistical significance was defined as a *P* value less than 0.05, at a 95% confidence interval (CI) not including 1.

## Results

### Patient characteristics based on A20 expression

This study included 442 patients, with a median age of 47 (22–86) years. Of the 442 patients, 390 (88.2%) had low A20 expressing tumors, and 52 (11.8%) had high A20 expressing tumors. Clinical characteristics were compared based on A20 expression in Table 1. High A20 expression was associated with anatomical tumor stage; A20 was highly expressed in advanced T stage (*P* = 0.006), N stage (*P* = 0.045), and AJCC stage tumors (*P* = 0.030). In addition, high A20 expression was associated with younger age at surgery (*P* = 0.011). However, it was not related to tumor grade (*P* = 0.299) or receptor status (ER; *P* = 0.765, PR; *P* = 0.412, HER2; *P* = 0.837). In addition, there was no statistical difference in adjuvant treatment, such as chemotherapy (*P* = 0.724), radiotherapy (*P* = 0.763) or endocrine therapy (*P* = 0.288).

**Table 1:** Clinical characteristics according to A20 expression.

	A20-low, n = 390 (%)	A20-high, n = 52 (%)	*P* value
**Age**			0.011
>35	355(91.0)	41(78.8)	
≤35	35(9.0)	11(21.2)	
**T stage**			0.006
1	141(36.2)	13(25.0)	
2	230(59.0)	30(57.7)	
3	18(4.6)	8(15.4)	
4	1(0.3)	1(1.9)	
**N stage**			0.045
0	219(56.2)	19(37.3)	
1	111(28.5)	19(37.3)	
2	33(8.5)	6(11.8)	
3	27(6.9)	8(15.4)	
AJCC stage*			0.030
I	133(34.1)	12(23.1)	
II	188(48.2)	24(43.2)	
III	67(17.2)	14(26.9)	
IV	2(0.5)	2(3.8)	
**HG**^**1**^			0.299
I, II	204(52.3)	33(63.5)	
III	164(42.1)	19(36.5)	
**ER**^**1**^			0.765
Positive	172(44.1)	21(40.4)	
Negative	216(55.4)	30(57.7)	
**PR**^**1**^			0.412
Positive	172(44.1)	24(46.2)	
Negative	216(55.4)	27(51.9)	
**HER-2**^**1**^			0.837
Positive	65(16.7)	9(17.3)	
Negative	248(63.6)	31(59.6)	
**Subtype**^**1**^			0.503
Luminal/HER2(-)	108(27.7)	17(32.7)	
HER2(+)	117(30)	15(28.8)	
TNBC	140(35.9)	14(26.9)	
**Operation method**			0.760
Total mastectomy	253(64.9)	35(67.3)	
Breast conserving	137(35.1)	17(32.7)	
**LVI**^**1**^			0.079
Positive	44(11.3)	11(21.2)	
Negative	315(80.8)	40(76.9)	
**Chemotherapy**			0.724
Done	303(77.7)	39(75.0)	
Not done/unknown	87(22.3)	13(25.0)	
**Radiotherapy**			0.763
Done	154(39.5)	19(36.5)	
Not done/unknown	236(60.5)	33(63.5)	
**Endocrine therapy**			0.288
Done	153(39.2)	16(30.8)	
Not done/unknown	237(60.8)	36(69.2)	
**Trastuzumab**			0.235
Done	15(3.8)	0	
Not done/unknown	375(96.2)	52(100)	

ER, estrogen receptor; PR, progesterone receptor; HER-2, human epidermal growth factor receptor-2; HG, histologic grade; TNBC, triple negative breast cancer; LVI, Lymphovascular invasion

*AJCC stage was performed based on 7^th^ edition

^1^Missing value

^2^HER-2 positive was defined by 3 positive on immunohistochemistry or amplification on fluorescence *in situ* hybridization

We classified patients into three subtypes based on ER, PR, and HER2 status; 125 patients were of the luminal/HER2-negative subtype, 132 were of the HER2-positive subtype, and 154 were of the TNBC subtype. There were no significant associations between A20 expression and breast cancer subtype (*P* = 0.503).

### Prognostic impact of A20 expression

At a median follow-up time of 92.5 months (1–258), 88 mortality events had occurred. There were a total of 110 recurrence events, of which 29 were locoregional recurrences, 97 were distant metastases, and 16 were both locoregional recurrences and distant metastases. Of 110 recurrence cases, there were 72 mortality events from breast cancer.

**Fig 1:**
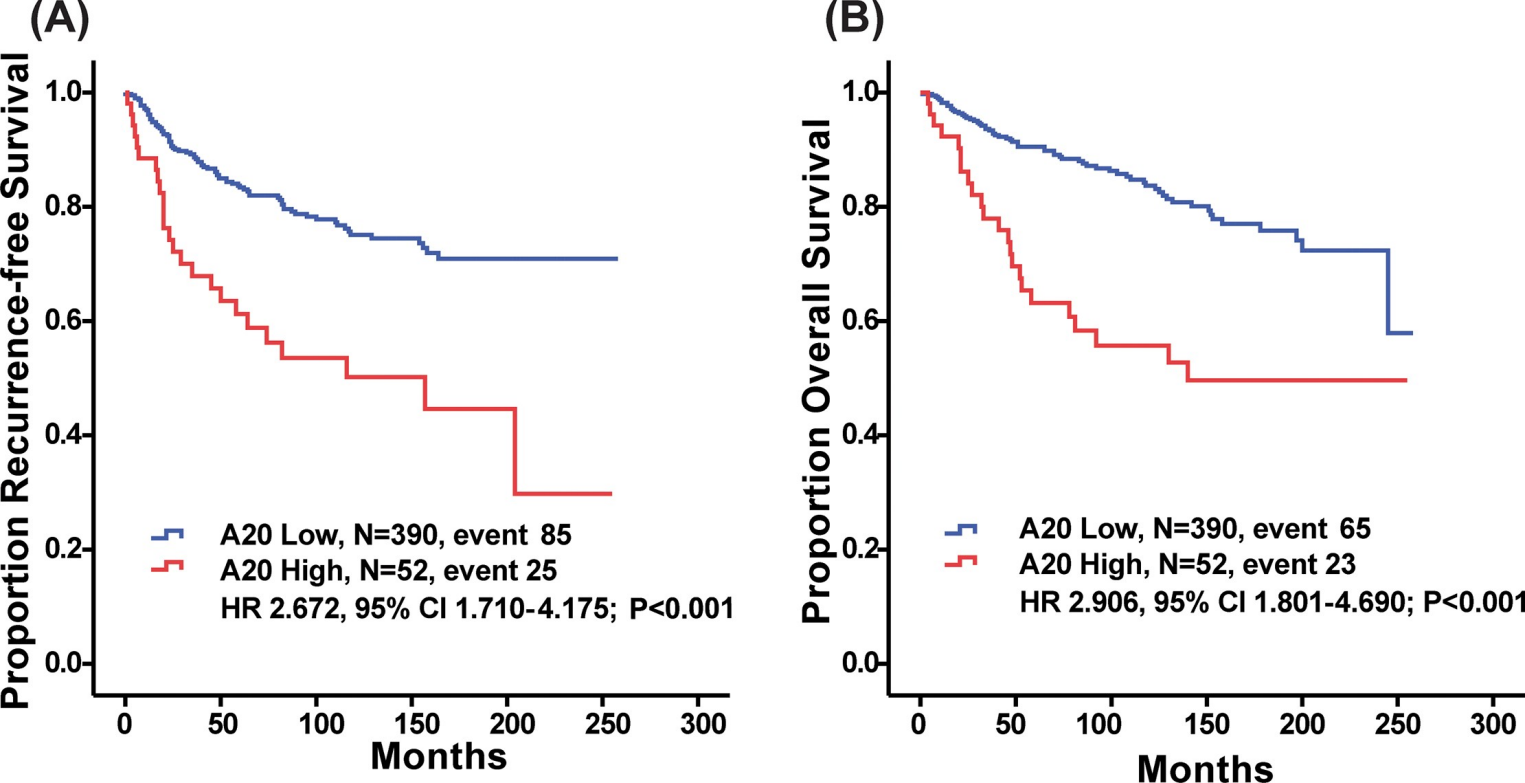
Kaplan-Meier plots of recurrence free survival (RFS) and overall survival (OS) according to A20 expression level. (a) RFS (A20 Low, M = 390, event 85; A20 High, N = 52, event 25; HR 2.672, 95% CI 1.710–4.175; *P*<0.001, respectively, log-rank test) and (b) OS differed significantly according to A20 expression level (A20 Low, N = 390, event 65; A20 High, N = 52, event 23, HR 2.906, 95% CI 1.801–4.690; *P*<0.001).

High A20 expression was significantly predictive of decreased RFS (*P* < 0.001, log-rank test; **Fig 1A**). Patients with high A20 expression also showed reduced OS (*P* < 0.001, log-rank test; **Fig 1B**). Younger age at surgery (≤35 years old), advanced T stage, advanced N stage, LVI and A20 (HR 2.672, 95% CI 1.710–4.175, *P*<0.001) were significant factors in the univariate analysis of RFS (Table 2). A20 (HR 2.906, 95% CI 1.801–4.690, *P*<0.001) was still a significant variable in the univariate analysis of OS (Table 3), too.

**Table 2:** Hazard ratios (HRs) and 95% confidential intervals (CIs) for recurrence free survival (RFS).

	Univariate analysis		Multivariate analysis	
	HRs (95% CI)	*P* value	HRs (95% CI)	*P* value
**Age**		<0.001		<0.001
>35	1		1	
≤35	0.355(0.227–0.555)		0.416(0.256–0.674)	
**T stage**		0.001		0.514
1	1		1	
2	1.5661.005–2.442)	0.048	1.293(0.801–2.085)	0.293
3	4.196(2.162–8.142)	<0.001	1.740(0.833–3.635)	0.141
**N stage**		<0.001		<0.001
0	1		1	
1	1.694(1.066–2.690)	0.026	1.339(0.809–2.214)	0.256
2	3.702(2.111–6.492)	<0.001	2.938(1.601–5.393)	0.001
3	5.313(3.057–9.232)	<0.001	3.969(2.068–7.619)	<0.001
**LVI**		<0.001		0.272
Negative	1		1	
Positive	2.377(1.468–3.848)		1.349(0.791–2.300)	
**HG**		0.990		
I and II	1			
III	1.001(0.825–1.216)			
**A20**		<0.001		<0.001
Low	1		1	
High	2.672(1.710–4.175)		2.324(1.446–3.736)	
**ER**		0.535		
Negative	1			
Positive	1.127(0.772–1.646)			
**PR**		0.535		
Negative	1			
Positive	0.887(0.607–1.296)			
**HER2**		0.862		
Negative	1			
Positive	0.954(0.560–1.624)			
**TNBC vs non-TNBC**		0.842		
non-TNBC	1			
TNBC	1.042(0.692–1.570)			

LVI, Lymphovascular invasion; HG, histologic grade; ER, estrogen receptor; PR, progesterone receptor; HER-2, human epidermal growth factor receptor-2; TNBC, triple negative breast cancer

**Table 3:** Hazard ratios (HRs) and 95% confidential intervals (CIs) for Overall survival (OS).

	Univariate analysis		Multivariate analysis	
	HR (95% CI)	*P* value	HR (95% CI)	*P* value
**Age**		0.001		0.042
>35	1		1	
≤35	0.429(0.260–0.708)		0.536(0.325–0.978)	
**T stage**		<0.001		0.073
1	1		1	
2	1.677(0.995–2.828)	0.052	1.340(0.763–2.355)	
3	5.751(2.836–11.662)	<0.001	2.123(0.955–4.718)	
**N stage**		<0.001		<0.001
0	1		1	
1	1.460(0.845–2.524)	0.175	1.128(0.619–2.053)	
2	3.847(2.086–7.093)	<0.001	2.894(1.483–5.648)	
3	6.566(3.672–11.741)	<0.001	4.067(2.027–8.162)	
**LVI**		<0.001		0.116
Negative	1		1	
Positive	3.079(1.833–5.172)		1.595(0.891–2.856)	
**HG**		0.492		
I and II	1			
III	1.080(0.867–1.344)			
**A20**		<0.001		<0.001
Low	1		1	
High	2.906(1.801–4.690)		2.629(1.585–4.361)	
**ER**		0.292		
Negative	1			
Positive	0.793(0.515–1.221)			
**PR**		0.330		
Negative	1			
Positive	0.808(0.526–1.241)			
**HER2**		0.831		
Negative	1			
Positive	0.934(0.497–1.754)			
**TNBC vs non-TNBC**		0.857		
Non-TNBC	1			
TNBC	1.045(0.645–1.693)			

LVI, Lymphovascular invasion; HG, histologic grade; ER, estrogen receptor; PR, progesterone receptor; HER-2, human epidermal growth factor receptor-2; TNBC, triple negative breast cancer

When adjusted for age, T stage, N stage, and LVI using the Cox proportional hazards regression model, high A20 expression was significantly associated with RFS and OS (RFS; Table 2, HR 2.324, 95% CI 1.446–3.736, *P*<0.001, Table 3; OS, HR 2.629, 95% CI 1.585–4.361, *P*<0.001).

To evaluate an improvement of predicting ability between multivariate models with and without A20, we calculated c-index, NRI, and IDI. The addition of A20 to base model increased c-indices for both OS and RFS without a statistical significance (0.733 to 0.755 for OS; 0.689 to 0.698 for RFS; Table 4). At a median follow-up 93 months, the addition of A20 improved a discriminating ability for OS because NRI and IDI were 0.230 and 0.047 (*P* = 0.028 and *P* = 0.004, respectively; Table 4). At a median follow-up 83 months, a model with A20 showed a superior discrimination by IDI (0.030, *P* = 0.008) whereas a NRI for this model was greater than 0 (0.151) but not significant (*P* = 0.076).

**Table 4:** Evaluation of Cox proportional hazard model using Harrell’s C index, NRI and IDI.

	OS			RFS		
	Harrell’s C index	NRI^1^	IDI^2^	Harrell’s C index	NRI	IDI
**Base model**	0.733(0.673–0.789)			0.689(0.630–0.748)		
**Base model +A20**	0.755(0.700–0.807)	0.230(0.051–0.380)	0.047(0.010–0.096)	0.698(0.639–0.755)	0.151(-0.034–0.261)	0.030(0.004–0.067)
***P* value**	0.169	0.028	0.004	0.413	0.076	0.008

NRI, net reclassification improvement; IDI, integrated discrimination improvement

### Prognostic impact of A20 expression in aggressive subtypes

**Fig 2:**
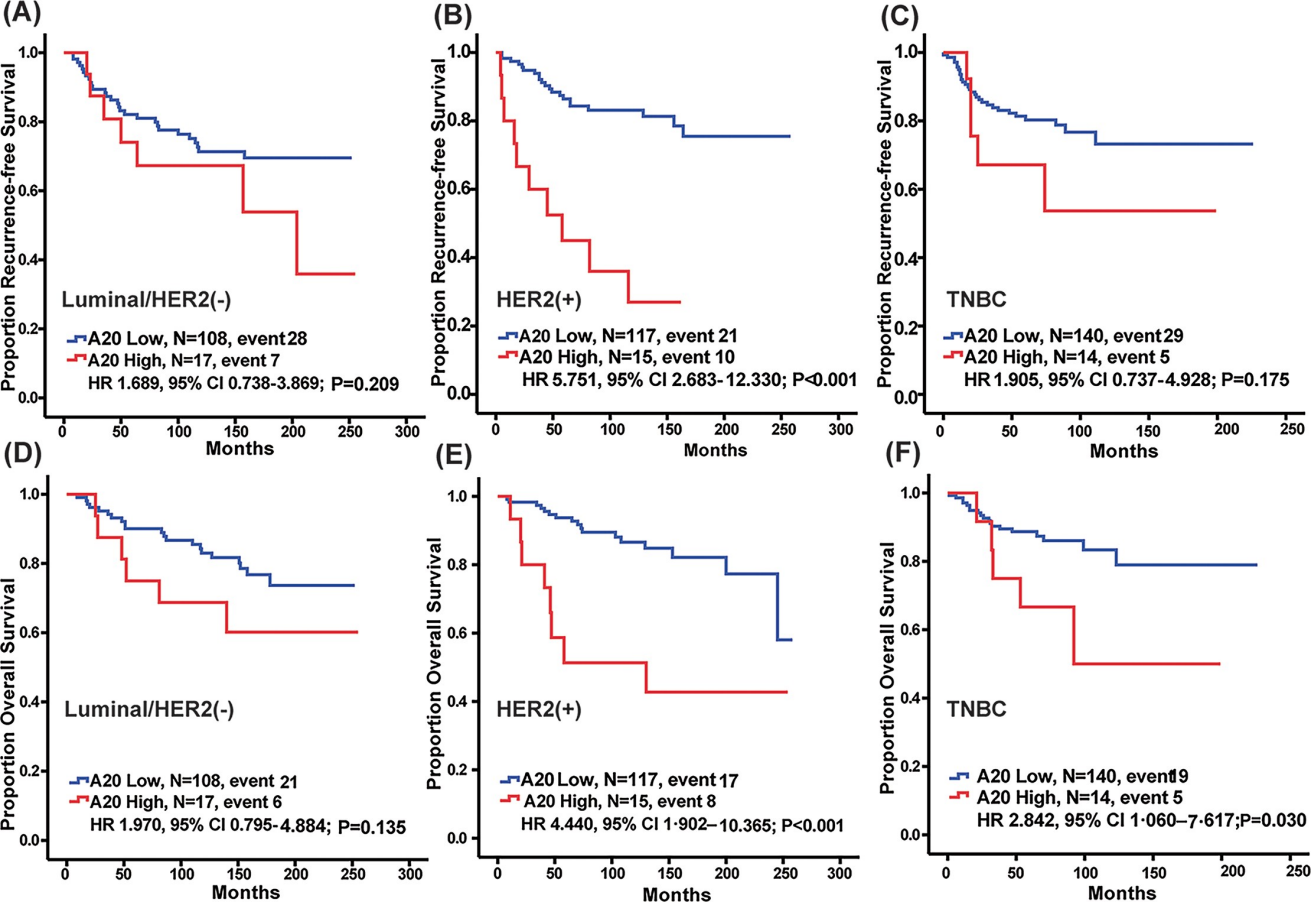
Kaplan-Meier plots of RFS and OS according to A20 expression level in each subtype. RFS differed significantly according to A20 expression in the HER2 subtype (Fig B; HR 5.751, 95% CI 2.683–12.330, P < 0.001, respectively, log-rank test), but it did not differ in the other subtypes (Fig A, Luminal/HER2 negative, HR 1.689, 95% CI 0.738–3.869, P = 0.209; Fig C, TNBC, HR 1.905, 95% CI 0.737–4.928, P = 0.175). OS differed significantly according to A20 expression in the HER2 and TNBC subtypes (Fig E; HER2, HR 4.440, 95% CI 1.902–10.365, P < 0.001, Fig 2F; TNBC, HR 2.842, 95% CI 1.060–7.617, P = 0.030), but it did not differ in luminal/HER2 negative (Fig D; HR 1.970, 95% CI 0.795–4.884, P = 0.135).

Survival outcomes based on A20 expression were compared in each breast cancer subtype. RFS differed significantly according to A20 expression in the HER2 subtype (Fig 2B; HR 5.751, 95% CI 2.683–12.330, *P* < 0.001), but it did not differ in the other subtypes (Fig 2A, Luminal/HER2 negative, HR 1.689, 95% CI 0.738–3.869, *P* = 0.209; Fig 2C, TNBC, HR 1.905, 95% CI 0.737–4.928, *P* = 0.175). In addition, decreased OS was noted in the high A20-expressing HER2-positive subtype (Fig 2E; HR 4.440, 95% CI 1.902–10.365, *P* < 0.001) and TNBC subtypes subtype (Fig 2F; HR 2.842, 95% CI 1.060–7.617, *P =* 0.030) of breast cancer, but it was not observed in the luminal/HER2-negative subtype (Fig 2D; HR 1.970, 95% CI 0.795–4.884, *P =* 0.135).

Univariate analysis of OS and RFS in each subtype was performed using the Cox-regression model. In HER2-positive subtype, A20 expression was a significant factor for prediction of both OS (Table 5; HR 3.987, 95% CI 1.628–9.760, *P =* 0.002) and RFS (Table 5; HR 5.276, 95% CI 2.395–11.620, *P<*0.001). For the TNBC subtype, A20 expression was a significant factor for prediction of OS (Table 5; HR 2.842, 95% CI 1.060–7.617, *P =* 0.038).

**Table 5:** Univariate analysis on A20 in each subtype.

	OS		RFS	
	HR (95% CI)	*P* value	HR (95% CI)	*P* value
**Luminal/HER2 negative**	1.970(0.795–4.884)	0.143	1.689(0.738–3.869)	0.215
**HER2-positive**	3.987(1.628–9.760)	0.002	5.276(2.395–11.620)	<0.001
**TNBC**	2.842(1.060–7.617)	0.038	1.905(0.737–4.927)	0.184

HR, hazard ratio; CI, confidential interval; HER-2, human epidermal growth factor receptor-2; TNBC, triple negative breast cancer

## Discussion

In this study, we found that A20 expression was an independent prognostic factor for breast cancer. Compared to patients with low A20-expressing tumors, patients with high A20-expressing tumors showed poorer RFS and OS (RFS, HR: 2.324, 95% CI: 1.446–3.736; OS, HR: 2.629, 95% CI: 1.585–4.361). Although A20 expression was not associated with specific subtypes, the prognostic impact of A20 expression was more pronounced in aggressive subtypes, such as HER2-positive and TNBC subtypes. It might be implicated that A20 has clinical value as a novel target for improving treatment outcomes in patients with aggressive breast cancer, such as HER2-positive subtype.

Earlier studies on A20 in inflammatory and autoimmune diseases showed that A20 is associated with a complex regulator of ubiquitylation of canonical NF-κB and cell-survival signals A20: linking a complex regulator of ubiquitylation to immunity and human disease [2–10, 21, 22]. In contrast, the role of A20 in malignant disease has not been extensively explored. Several genetic studies have reported that deletion or mutation of A20 is associated with hematologic malignancies, such as multiple lymphoma, MALT lymphoma, Hodgkin’s disease, and follicular lymphoma, which are characterized by NF-κB signal activation [23–26]. Other studies have suggested that high A20 expression was associated with tumor survival and growth in solid tumors such as malignant glioma and breast cancer [11–14]. Previous studies on the role of A20 in malignancies suggest that it acts as a tumor suppressor or enhancer depending on the cell type.

In addition to the above findings, A20 has also been reported to have a novel role in breast cancer; A20 promotes tumor progression and activates EMT signals, mainly by affecting SNAIL1 stabilization [16]. EMT signaling, which is associated with the capacity to migrate to distant organs [15], is frequently activated in TNBC [27, 28]. A mechanism by which the EMT pathway is activated via A20 elevation [16] might partly explain our findings: poor survival outcomes in TNBC patients with high A20 expression. Further studies of EMT features, such as high vimentin expression or E-cadherin loss, in TNBC with high A20 expression levels are warranted.

Intriguingly, we also found that high A20 expression is a poor prognostic marker in patients with HER2-positive breast cancer. Several studies have provided evidence that upregulation of NF-κB signaling in HER2-positive breast cancer is associated with resistance to various therapies, including anti-HER2 therapy [29, 30]. Although A20 is a negative regulator of NF-κB signaling in inflammation and immune response, a previous study by Lerebours et al. showed that A20 is involved in upregulation of the NF-κB pathway in inflammatory breast cancer (IBC) [31]. Although HER2 status was not reported in the study, it has been noted that 30~50% of IBCs have HER2 overexpression [32–34], suggesting that A20 expression might be associated with NF-κB pathway activation in HER2-positive breast cancer. Further studies on the association between A20 and NF-κB are warranted in HER2-positive cancer.

The biology underlying poor outcomes in patients with high A20-expressing HER2-positive breast cancer is also supported by a mechanism of A20-mediated TGF-β-activation. Previous studies have shown that HER2 stimulates TGF-β-mediated EMT, contributing to the aggressive behavior of HER2-positive breast cancer [35–37]. Our previous study also provided evidence that A20 elevates TGF-β signaling, suggesting that A20 could promote a TGF-β-meditated EMT pathway and tumor aggressiveness in HER2-positive breast cancer.

Other studies have reported that A20 overexpression was associated with endocrine therapy, chemotherapy, and radiotherapy treatment failure. A preclinical study of MCF7 cells demonstrated that tamoxifen-resistant cancer cells showed A20 overexpression [14]; however, there was no prognostic influence of A20 expression in patients with luminal/HER2-negative breast cancer. In addition, another study suggested that A20 reduced the efficacy of radiotherapy and chemotherapy via the regulation of anti-apoptotic mechanisms [38].

Our study had several limitations. First, it was a retrospective study with a small sample size, and the enrolled patients received uncontrolled adjuvant treatments. Particularly, most patients (88.5%) with HER2-positive tumors did not receive anti-HER2 therapy (S2 Fig) because most of the HER2-positive samples were collected prior to 2010 when the use of trastuzumab was not reimbursed by the national insurance system in Korea. In addition, there were higher proportions of HER2-positivie and TNBC subtypes in our study than there are in global breast cancer populations, because of selection bias during sample storage and preservation. Despite these limitations, our study comprehensively showed the clinical outcomes based on A20 expression among patients with breast cancer and demonstrated A20 expression as an independent prognostic factor. Furthermore, prognostic discrimination based on A20 expression was pronounced in aggressive tumors, such as HER2-positive and TNBC subtypes. Our findings suggest that A20 may be a valuable target in patients with aggressive breast cancer.

## Conclusion

In conclusion, we found that A20 expression is a poor prognostic marker in breast cancer. The prognostic impact of A20 was pronounced in aggressive tumors, such as HER2-positive and TNBC subtypes. Our findings suggested that A20 may be a valuable target in patients with aggressive breast cancer

## Supporting information

S1 Fig Immunohistochemical analysis of A20 expression. A20 expression was evaluated in high-power fields (400× magnification) by an experienced pathologist (A.O.) (a) Negative for A20. (b) 1+ for A20. (c) 2+ for A20. (d) 3+ for A20.
(TIF)

S2 Fig Kaplan-Meier plots of RFS and OS for HER2 subtype breast cancer according to trastuzumab use. (a) RFS and (b) OS did not differ significantly according to trastuzumab use (*P* = 0.050 and *P* = 0.102, respectively, log-rank test).
(TIF)
