# Supplementary figures and images for: High A20 expression negatively impacts survival in patients with breast cancer
Source: PLoS One. 2019 Aug 26;14(8):e0221721. doi: 10.1371/journal.pone.0221721 (PMC6709902; doi:10.1371/journal.pone.0221721)

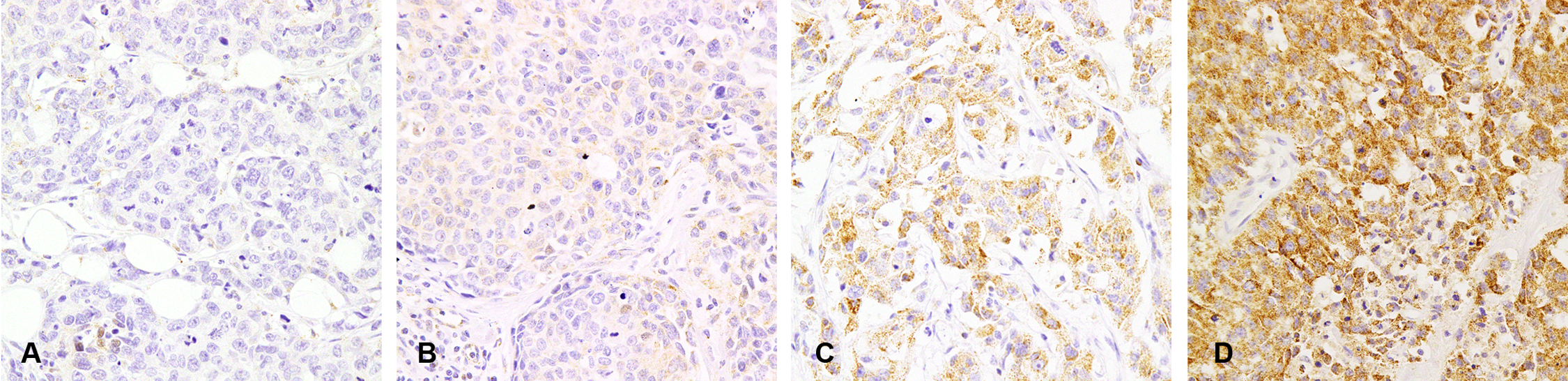

Supplement: S1 Fig — A20 expression was evaluated in high-power fields (400× magnification) by an experienced pathologist (A.O.) (a) Negative for A20. (b) 1+ for A20. (c) 2+ for A20. (d) 3+ for A20. (TIF) [file pone.0221721.s001.tif]

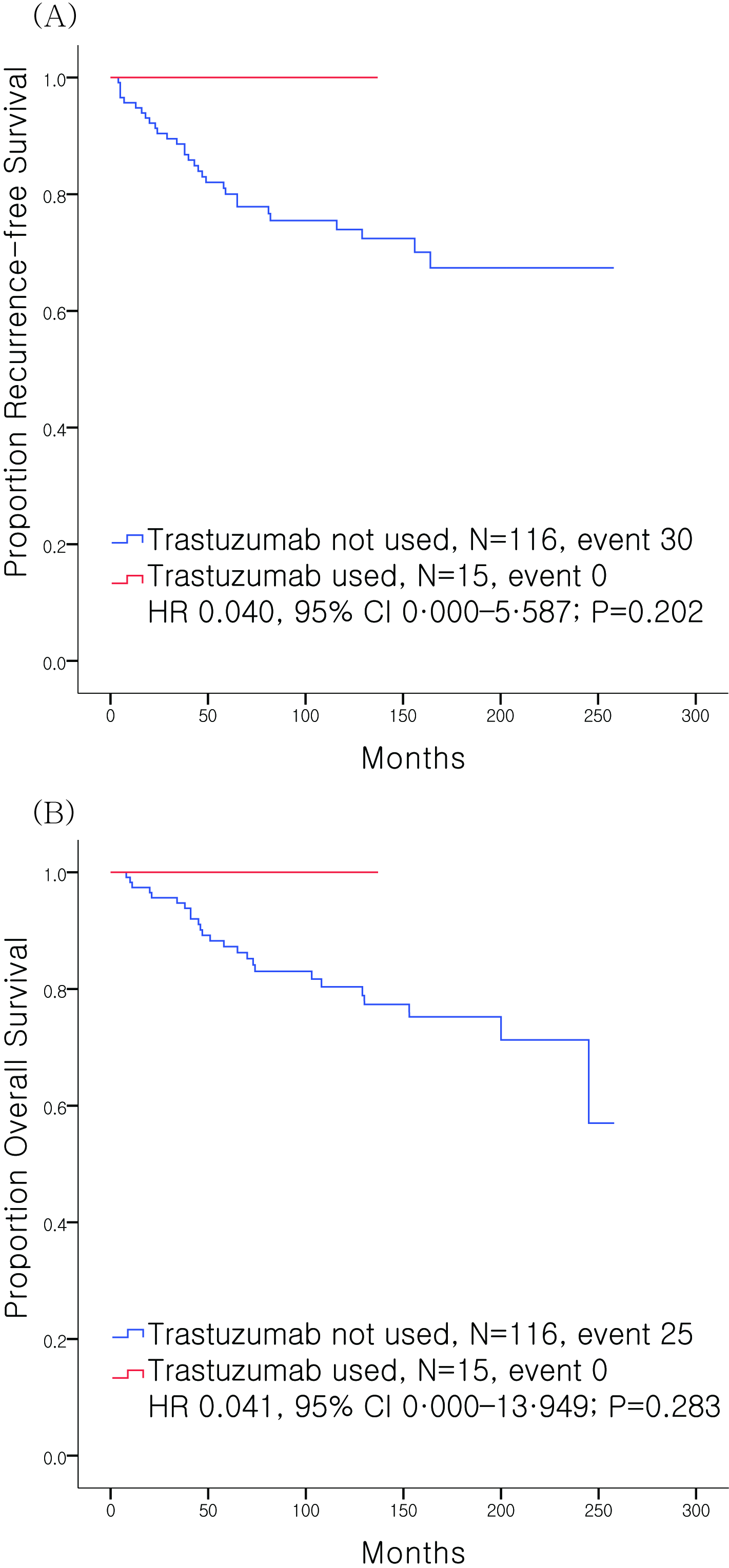

Supplement: S2 Fig — (a) RFS and (b) OS did not differ significantly according to trastuzumab use (P = 0.050 and P = 0.102, respectively, log-rank test). (TIF) [file pone.0221721.s002.tif]
